# Interactive roles of chromatin regulation and circadian clock function in plants

**DOI:** 10.1186/s13059-019-1672-9

**Published:** 2019-03-22

**Authors:** Z. Jeffrey Chen, Paloma Mas

**Affiliations:** 1 Department of Molecular Biosciences, The University of Texas at Austin, Austin, TX 78712 USA; 2 Department of Integrative Biology, The University of Texas at Austin, Austin, TX 78712 USA; 3 Center for Research in Agricultural Genomics (CRAG), Consortium CSIC-IRTA-UAB-UB, Campus UAB, Bellaterra, 08193 Barcelona, Spain; 4 Consejo Superior de Investigaciones Científicas, 08028 Barcelona, Spain

## Abstract

Circadian rhythms in transcription ultimately result in oscillations of key biological processes. Understanding how transcriptional rhythms are generated in plants provides an opportunity for fine-tuning growth, development, and responses to the environment. Here, we present a succinct description of the plant circadian clock, briefly reviewing a number of recent studies but mostly emphasizing the components and mechanisms connecting chromatin remodeling with transcriptional regulation by the clock. The possibility that intergenomic interactions govern hybrid vigor through epigenetic changes at clock loci and the function of epialleles controlling clock output traits during crop domestication are also discussed.

## Introduction

The Earth’s rotation around its axis leads to changes in light and temperature that have shaped life over evolution. It is therefore not surprising to find 24-h rhythms in physiology, metabolism, and development that oscillate in synch with the day and night cycles [1, 2]. A robust and yet flexible cellular machinery, the circadian clock, generates the rhythms by integrating the environmental cues and the temporal information into 24-h biological oscillations [1, 2]. As sessile organisms, plants must effectively perceive and appropriately respond to the changes in environmental conditions for proper growth and survival [3, 4]. Consistently, it has become increasingly clear that the circadian clock controls the phase of a vast collection of pathways in plants.

A highly precise circadian clock function is crucial for proper plant adaptation to the environment [5]. Genome-wide analyses have provided evidence of the pervasive role of the clock controlling the rhythms of a large fraction of the transcriptome [6–11]. The rhythms in gene expression are transduced into oscillations of protein activities involved in a myriad of signaling pathways. Germination, growth, development [12–15], and responses to abiotic [16, 17] and biotic [18, 19] stresses are just a few of the many examples of processes controlled by the plant circadian clock. Recent studies have expanded the range of the pathways controlled by the clock. Indeed, the repertoire of circadianly regulated processes also includes the regulation of other oscillators such as the cell cycle. The study showed that circadian control of the cell cycle is exerted by setting the time of DNA replication licensing [20]. Similarly, another recent study has shown that the circadian clock regulates age-dependent and dark-induced leaf senescence [21, 22]. The mechanisms rely on the clock-controlled regulation of the positive aging regulator *ORESARA1* (*ORE1*) [21, 22] and on the repression of miR164, a post-transcriptional repressor of ORE1 [21]. Leaf senescence also relies on the function of circadian clock components that gate the signaling of the phytohormone jasmonate [23]. Overall, the circadian clock ensures proper phasing of these biological processes in consonance with the environment. The clock function thus requires precise information on the environmental fluctuations. This occurs through the activity of photoreceptors that perceive and transduce light and temperature changes. Hence, the clock machinery exploits photoreceptor function for time-of-day information [24]. Resetting of the clock by these environmental changes is assumed to occur through changes in the expression and activity of essential clock components [25].

## Components and regulatory mechanisms of circadian clock activity in *Arabidopsis*

The main *Arabidopsis* clock components entangle in a complex regulatory network that generates rhythms in expression and activity exerted at specific phases during the day and night [26]. Briefly, the morning-expressed and partially redundant single MYB transcription factors known as CCA1 (CIRCADIAN CLOCK ASSOCIATED1) [27] and LHY (LATE ELONGATED HYPOCOTYL) [28] repress the expression of the evening-expressed clock genes during the day [26]. In turn, evening-expressed clock components such as TOC1/PRR1 (TIMING OF CAB2 EXPRESSION1/PSEUDO RESPONSE REGULATOR1) [29, 30] repress the morning genes during the night [31–33]. TOC1 belongs to a family of clock repressors (including PRR9, PRR7, PRR5, and PRR3 in addition to TOC1) that sequentially suppress *CCA1* and *LHY* transcription during the day [34]. Repression starts with PRR9 function at early midday and is subsequently followed by PRR7 and PRR5 later in the day [34] and by TOC1 at dusk and early evening [31–33]. TOC1 represses not only *CCA1* and *LHY* expression but also nearly all of the oscillator components [35]. Other evening-expressed regulators, including LUX (LUX ARRYTHMO), ELF3 (EARLY FLOWERING3), and ELF4 (EARLY FLOWERING4), form a protein complex (evening complex) that acts as a repressor of the morning-expressed *PRR* clock genes [36–40]. Repression of the *PRR* genes by evening complex permits the rising phase of *LHY* and *CCA1*, which reach their peak expression at dawn.

In addition to this battery of clock repressors, direct activation of circadian gene expression relies on the function of the single MYB REVEILLE/LHY-CCA1-LIKE (RVE/LCL) transcription factors, which share a high sequence homology with CCA1 and LHY, particularly in the MYB domain [41]. RVE8, RVE6, and RVE4 directly interact with the clock-related components known as LNKs (NIGHT LIGHT-INDUCIBLE AND CLOCK-REGULATED) to activate the expression of clock genes such as *TOC1* and *PRR5* [42–47]. The mechanisms of regulation rely on changes in chromatin modifications [42] and recruitment of the basal transcriptional machinery to the circadian loci [48]. Additional key clock components and post-transcriptional and post-translational mechanisms of regulation ensure smooth shapes of the oscillatory waves, fine-tuning the robustness and precision of the clock. Altogether, the complex regulatory circadian network at the core of the clock ensures that the morning and evening clock transcripts precisely peak at their corresponding phases [26]. It was recently proposed that the complexity of the plant circadian network might provide strength against extreme environmental conditions [49].

Long-standing questions in plant circadian biology deal with how the circadian clocks are organized within the plant body and whether there are overarching signals that synchronize the clocks in separate parts of the plant. Nearly all cells possess clocks exhibiting various degrees of synchronization. Early studies reported that different rhythmic oscillations could be controlled by separate oscillators [50] and that autonomous clocks were able to regulate gene expression [51] in a tissue-specific manner [52, 53]. Despite the organ-specific synchronization [54], long-distance signals are important for clock synchronization in distal parts of the plant [55, 56]. Short-distance communication or circadian coupling also plays a role in synchronization. The degree of coupling varies depending on tissues and conditions. For instance, cells at the vasculature present stronger coupling than leaf cells [57, 58], which show only weak coupling [59–61]. In root cells, a continuous resetting of the circadian oscillations results in a stripe wave originating at the root tip [62], which shows strong cell-to-cell coupling [63]. Gould et al. [63] proposed that the variability in coupling and period differences among different root cells can explain the waves of clock activity in roots. Synchronization in roots can also occur by light piping from shoots [64]. The shoot apex represents a particular example of short- and long-distance circadian communication, as rhythms at the shoot apex are highly synchronized due to strong circadian coupling, and this function is important for proper rhythms in roots [56].

## Chromatin remodeling and transcriptional regulation

Transcriptional rhythms underlie the circadian clock function at its basis. As transcriptional regulation is largely dependent on chromatin status, understanding changes in chromatin conformation is essential to fully comprehend rhythms in transcription. Chromatin can be modified at levels of DNA sequence, histones, and high-order chromatin structure and organization [65–67]. DNA methylation affects growth and development of plants and animals in response to environmental cues [68–71] and is essential for animal development [72]. Plants are more tolerant to mutations in DNA methylation pathways [68, 70], and methylation mutants have few phenotypes, although abnormal genetic lesions can develop over several generations of self-pollination [73]. Unlike in animals in which methylation occurs almost exclusively in the CG context [74], with a few exceptions in stem cells [75], methylation in plants occurs in CG, CHG, and CHH (H = A, T or C) contexts through distinct pathways [71]. In *Arabidopsis*, METHYLTRANSFERASE 1 (MET1) and CHROMOMETHYLASE 3 (CMT3) are responsible for the maintenance of CG and CHG methylation, respectively [76–78]. CHH methylation is established de novo through two pathways. One involves biogenesis of small interfering RNAs (24-nt siRNAs) that require Nuclear RNA Polymerase IV (D) Subunit1 (NRPD1) [79, 80] and are targeted to corresponding genomic loci by ARGONAUTE (AGO) family members (AGO4 and AGO6), which are methylated via DOMAINS REARRANGED METHYLTRANSFERASE2 (DRM2) [81, 82]. The other pathway requires CHROMOMETHYLASE 2 (CMT2) through interacting with DECREASE IN DNA METHYLATION1 (DDM1) in histone H1-containing heterochromatic regions [83]. In addition to its establishment and maintenance, DNA methylation can be actively removed by a family of bifunctional methyl-cytosine glycosylases-apurinic/apyrimidinic lyases through a base excision repair pathway [71]. These demethylases consist of REPRESSOR OF SILENCING 1 (ROS1) [84], DEMETER (DME) [85, 86], and DEMETER-LIKE 2 and 3 (DML2 and DML3) [87, 88]. DNA methylation may change gene expression, inducing imprinting and activation of transposable elements (TEs) and TE-associated genes, in response to developmental and environmental cues [71].

In addition to DNA methylation, the accessibility of chromatin is dynamically regulated by a suite of histone modifications, dubbed “histone code” [66]. Core histones (H2A, H2B, H3, and H4) can be covalently modified at different positions of amino-terminal tails by different modifications, including acetylation, methylation, ubiquitination, phosphorylation, glycosylation, carbonylation, ADP ribosylation, sumoylation, and biotinylation [66, 89, 90]. These modifications, alone or in combination, can change the accessibility of chromatin structures in the vicinity of genes to transcription machinery, leading to transcriptional activities and epigenetic phenomena [91]. Histone acetylation and deacetylation are reversible and controlled by histone acetyltransferases (HATs) as “writer” and histone deacetylases (HDACs) as “eraser” [89, 91, 92]. Most acetylation marks such as histone 3 lysine 9 acetylation (H3K9ac), histone 3 lysine 14 acetylation (H3K14ac), and histone 3 lysine 36 acetylation (H3K36ac) are associated with gene activation [89]. Plants have multiple gene families of HATs and HDACs [89, 93]. Plant HATs are grouped into two based on localization (nuclei or cytoplasm) [89] or five depending on sequence features [93]. The major class of HATs is the homologs of the GCN5 family in yeast and *Tetrahymena* [94]. Mutation of an *Arabidopsis AtGCN5* results in the reduction of histone H3 or H4 acetylation in the light-responsive promoter regions and reduced expression of the light-inducible genes [95]. Moreover, AtGCN5 interacts with CBF1 and mediates cold-inducible gene expression [96], which is regulated by the circadian clock [97].

Plants have homologs of histone deacetylases, including RPD3 (reduced potassium dependency protein3)-like and sir2-like (silent information regulator protein 2), which are conserved across all eukaryotes [89, 91]. In addition, plants have a specific histone deacetylase, HD2, which is identified in maize [98] and involved in gene repression and seed development in *Arabidopsis* [99]. RPD3-like HDACs, HDA19 or HD1, in *Arabidopsis* exhibit histone deacetylase activity [100] and are a general transcriptional regulator [101]. In the *athd1* mutant, approximately 7% of the genes are either up- or downregulated, while the upregulated genes are associated with elevated acetylation levels in a locus-specific manner [102]. HDA6, a homolog of HDA19, affects CG and CHG methylation and is involved in silencing of TEs and uniparental rRNA genes subjected to nucleolar dominance [91]. Yeast Sir2 is an NAD-dependent histone deacetylase and plays a role in transcriptional silencing and delayed aging [103]. Members of the SIRT family are associated with host–pathogen interactions in *Arabidopsis* [104], and DNA fragmentation and cell death in rice through changes in H3K9ac [105].

Like histone acetylation, histone methylation is reversible; but unlike histone acetylation, histone methylation can be associated with gene activation or repression depending on the site of modifications [90]. In general, histone H3 lysine 4 (H3K4) and H3K36 methylation is related to gene expression, while H3K9 and H3K27 methylation is related to gene repression and heterochromatin formation [65]. Histone methyltransferases (HMTs), as writers, are a group of proteins that contain SET (SU(VAR)/E(Z)TRX) domains to methylate histone H3 lysine residues. Plant SET domain proteins can be divided into four groups based on *Drosophila* members E(Z), TRX, AHS1, and SU(VAR)3-9 [106]. Some SET domain proteins belong to the members of Polycomb group (PcG) and regulate imprinting and gene expression during plant and animal development [107], while others are related to transcriptional activation and silencing [90, 91]. HMTs can have specificity for methylating lysine residues of histone H3. For example, SUVH4 (aka KRYPTONITE) is related to histone 3 lysine 9 mono/dimethylation (H3K9me1/2), *Arabidopsis* TRITHORX5 and 6 (ATX5 and ATX6) are associated with H3K27 methylation, and ASH 1 Homolog2 (ASHH2) mediates H3K36me2/3 methylation. SET Domain Group2 (SDG2) is a major writer for H3K4me1/2/3 and regulates plant growth and development [108], while ATX1 (SDG27) and ATX2 (SDG30) display locus-specific H3K42/3 methylation [109].

Histone demethylases or eraser proteins belong to two groups with distinct biochemical properties. Lysine-specific demethylase1 (LSD1) acts through amine oxidation, while a large family of Jumonji C (JmjC) domain-containing proteins directly reverse histone methylation by an oxidative demethylation process [110]. *Arabidopsis* has four LSD members and 21 JmjC homologs [111]; they play important roles in plant growth and development. JMJ5 (ELF6) and JMJ12 (relative early flowering6 (REF6)) promote early and late flowering phenotypes in their respective mutants [112]. Increase in Bonsai Methylation1 (IBM1) is a JmjC member (JMJ25) which counteracts H3K9 methylation, in addition to CHG DNA methylation, to prevent spreading of silencing from TEs and heterochromatin to active genes [113].

The interplay between histone acetylation, deacetylation, methylation, and demethylation is dynamic and interactive. For example, AtGCN5 and AtHDA19 are required for H3K36ac homeostasis. H3K36ac and histone 3 lysine 36 trimethylation (H3K36me3) show negative crosstalk, which is mediated by GCN5 and the histone methyl transferase SDG8 [114]. SUVH4 is a HMT for H3K9 methylation and interacts with CHROMOMETHYLASE 3 to maintain CHG DNA methylation at silenced loci [115]. When H3K9 and H3K27 methylation levels are high, these sites are void of acetylation. Silenced rDNA loci are de-repressed by 5-aza-2′-deoxycytidine (aza-dC), a chemical inhibitor for DNA methylation, and trichostatin A, a chemical inhibitor for histone deacetylation, suggesting interactive roles of DNA methylation and histone deacetylation in gene repression [116]. Moreover, other modifications of histones, such as phosphorylation and ubiquitination, may also contribute to cell cycle regulation and gene expression during plant development. Inhibition of histone deacetylation by trichostatin A in *Nicotiana sylvestris* protoplasts reduces H3S10ph at anaphase and telophase and induces the accumulation of metaphase cells [117].

Chromatin modification can also occur at levels of nucleosome remodeling and replacement of core histone with histone variants [91, 118]. DECREASE IN DNA METHYLATION1 (DDM1), encoding a SWI2/SNF2-like chromatin remodeling protein in plants, mediates DNA methylation and genome stability [119]. In *Arabidopsis*, histone variant H2A.Z is antagonistic with DNA methylation [120] and mediates thermosensory responses [121]; H2A.W marks the heterochromatin with H3K9 methylation [122]. Moreover, the three-dimensional structure in nuclei can also impact chromatin dynamics and spatial-temporal transcriptional regulation in animals [123] and possibly in plants.

Dynamic regulation of DNA methylation and chromatin modifications have been recognized to be essential for transcriptional regulation in response to growth and development in plants and animals [124]. The chromatin landscape is interwoven with circadian control of transcriptional regulatory networks with the corresponding spatial and temporal information [123].

## The interplay between chromatin remodeling and the *Arabidopsis* circadian clock

The first report describing a connection between chromatin remodeling and the *Arabidopsis* circadian clock uncovered a remarkable parallelism between the rhythmic changes in mRNA and the oscillatory pattern of histone 3 acetylation (histone 3 lysine 9 and 14 acetylation, H3K9/14ac) at the promoter of the clock gene *TOC1* [125]. The study also showed that repression of *TOC1* at dawn coincided with the binding of CCA1 to the *TOC1* promoter and with a hypo-acetylated state of H3. During the day, the *TOC1* mRNA rising phase correlates with increased H3ac that likely favors an open chromatin conformation, facilitating the accessibility of the transcriptional machinery and, hence, the transcription of the gene. Later studies showed that, during the day, the clock-related MYB transcription factor RVE8 contributes to the hyper-acetylated state of H3 at the *TOC1* promoter, antagonizing CCA1’s repressive function. The molecular mechanism by which RVE8 facilitates the increased H3ac was later identified [48] (see below). At the peak of *TOC1* expression, histone deacetylase activities contribute to the removal of acetyl groups from H3, leading to a hypo-acetylated state that correlates with the declining phase of *TOC1* mRNA [125]. In addition, *CHE*, another clock component, is repressed at ZT6 and ZT9 in the *AtHD1* mutant [126]. Further studies showed that CHE interacts with AtHD1 to repress *CCA1* expression; *CCA1* repression was relieved in the *che athd1* double mutant. The interaction of clock–chromatin proteins would add another layer of complexity in the circadian transcriptional feedback loop.

**Fig. 1:**
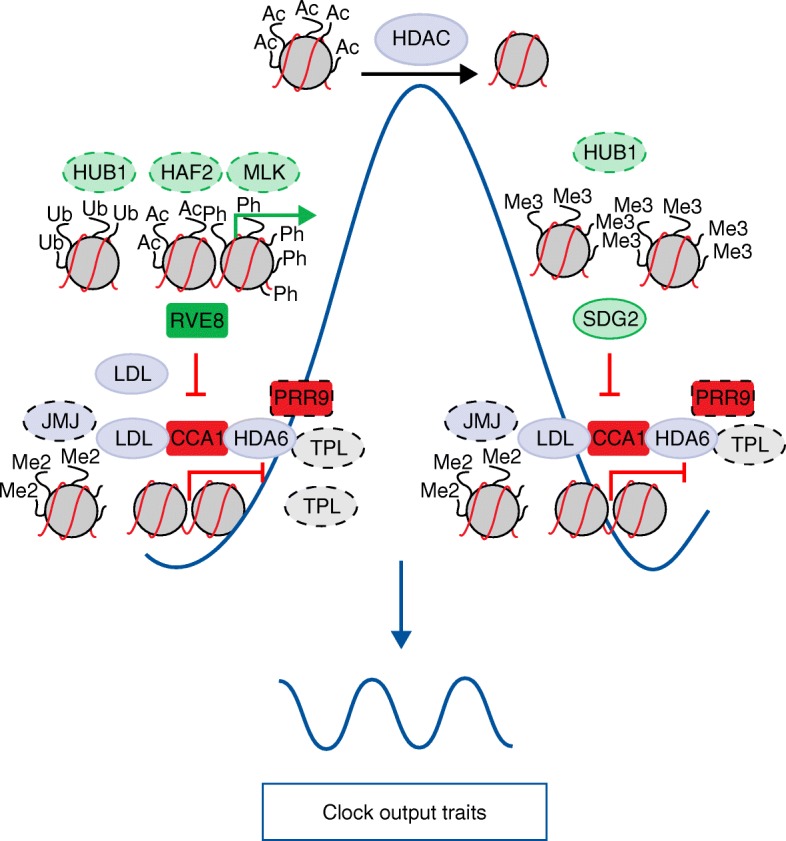
Main transcription factors (*rectangles*) and chromatin-related components (*ovals*) shaping the circadian waveform of clock gene expression. The rhythms in gene expression are transduced into oscillations of gene and protein activities involved in a myriad of clock output traits. The main factors regulating *TOC1* expression are shown: activators (*green*), repressors (*red*), and chromatin-related factors with a yet-to-be fully defined molecular function within the clock (*purple*). Components described to be involved in the regulation of other clock genes are also included (*ovals with dotted lines*). Further details are given in the text. Adapted from [125]

Other histone marks also associate with the chromatin state at the *TOC1* promoter [127–129]. For instance, histone 3 lysine 4 trimethylation (H3K4me3) accumulation is also rhythmic and peaks just after the peak of H3ac. The rhythms were observed under different photoperiods and under constant light conditions, suggesting a direct link with the circadian clock [127–129]. Accumulation of H3K4me3 antagonizes the binding of clock repressors such as CCA1, thus preventing repression from occurring too early and ensuring a precise 24-h rhythmic expression [129]. The histone methyltransferase SDG2/ATXR3 (SET DOMAIN GROUP 2/ARABIDOPSIS TRITHORAX RELATED 3) was proposed to contribute to the H3K4me3 accumulation as clock gene expression, H3K4me3 marks, and clock repressor binding were affected in plants miss-expressing SDG2/ATXR3 [129]. The oscillatory accumulation of these histone marks paralleling the rhythmic mRNA accumulation is not exclusive for *TOC1* but is also present at the promoters of other oscillator genes such as *CCA1*, *LHY*, *PRR9*, *PRR7*, and *LUX* [129]. It was proposed that these histone marks could provide the rhythmic activation necessary for expression, particularly in a circadian signaling network full of repressors [130] (Fig. 1). A recent report has further explored the connection between the clock and chromatin dynamics identifying a H3K9ac/H3K27ac/H3S28ph signature as a mechanism controlling diurnal transcript changes [131]. Baerenfaller et al. [131] proposed that H3K4me3 marks and the absence of the repressive H3K9me2 and histone 3 lysine 27 trimethylation (H3K27me3) might be responsible for the control of the steady active states.

**Fig. 2:**
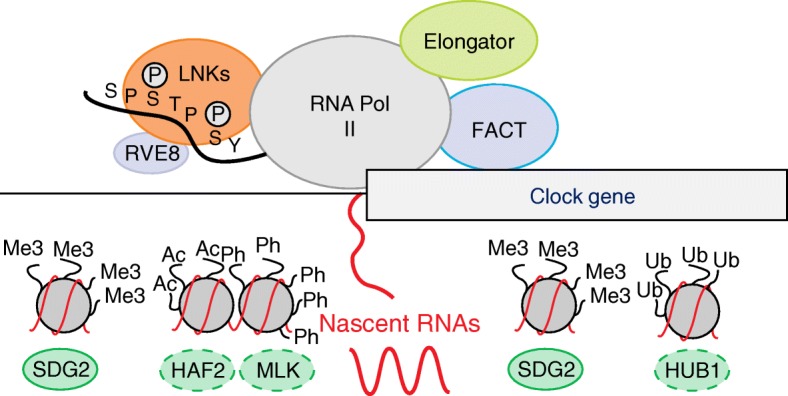
Protein complex formation, including clock proteins and the transcriptional machinery, controls the rhythms of chromatin modifications and nascent RNA of clock genes. Rhythmic binding of RVE8 (REVEILLE8) to the target clock promoters and its interactor LNKs (NIGHT LIGHT-INDUCIBLE AND CLOCK-REGULATE) facilitates the rhythmic recruitment of the transcriptional machinery, and the FACT (facilitates chromatin transcription) complex. This complex interplay facilitates oscillations in chromatin modifications and the rhythms of clock nascent RNAs. Further details are given in the text. Adapted from [48]

Another chromatin-activating function relies on HUB1 (HISTONE MONOUBIQUITINATION1), an unusual ubiquitin E3 ligase that is involved in histone H2B ubiquitination (H2Bub) [132]. Studies with *hub1-1* mutant plants showed a reduced amplitude in the expression of clock genes as well as in histone H2Bub and H3K4me3 marks associated with the gene coding regions [133]. These results together with the fact that H2Bub facilitates the function of the FACT (facilitates chromatin transcription) complex in humans [134] suggest a role for HUB1 on transcriptional elongation in plants [135]. It is noteworthy that the direct connection of the circadian clock with the FACT complex was previously hinted at [125] and later mechanistically confirmed [48]. Indeed, rhythms in transcript initiation and elongation of evening-expressed clock genes rely on the rhythmic recruitment of RNA polymerase II and the FACT complex to their promoters. The mechanism depends on the interaction of the clock-related components LNKs with RNA polymerase II and the FACT complex. In turn, the interaction of LNKs with RVE8, which is able to bind to the target promoters, allows the recruitment of the transcriptional machinery and associated chromatin remodeling complexes to rhythmically co-occupy the clock gene promoters [48]. This mechanism exemplifies an effective way for controlling chromatin status, transcript initiation and elongation, and proper rhythms in nascent RNAs [48]. These findings are consistent with a recent study showing that the expression of a subset of clock genes is downregulated in *elo* mutant plants [136]. These mutants are deficient in the elongator complex, which promotes RNA polymerase II-mediated transcript elongation through epigenetic activities such as histone acetylation [136] (Fig. 2).

Not just activating histone modifications are associated with the clock as other histone marks such as histone 3 lysine 36 dimethylation (H3K36me2) appear to negatively correlate with the expression of the oscillator genes [128]. Furthermore, the transcriptional repression of *CCA1* and *LHY* is regulated by members of the Groucho/Tup1 protein family, topless/topless-related (TPL/TPR), which interact with the PRR protein family at the *CCA1* and *LHY* promoters [137]. This repression is alleviated following treatment with the histone deacetylase inhibitor trichostatin A, suggesting that the histone deacetylase activity is required for TPL function. Also, PRR9, TPL, and HDA6 (histone deacetylase 6) form a protein complex likely involved in H3 deacetylation [137]. Therefore, TPL functions as an important chromatin-related repressor of core oscillator genes.

The clock seems in turn to feedback on chromatin regulation as the expression of a number of chromatin remodeling factors rhythmically oscillates [138]. Transcriptional regulation by the clock might be a way to temporally control the expression of the oscillator genes or other clock input or output genes. For instance, CCA1 directly binds to the promoter of *PKL* (*PICKLE*) [139]. The gene encodes an ATP-dependent chromatin remodeling factor that negatively regulates photomorphogenesis. Zha et al. [139] show that CCA1 regulation of *PKL* could be important for hypocotyl elongation under warm temperatures. It is interesting to note that the expression of *PKL* is downregulated in *cca1* mutant plants [139], which suggests that CCA1 activates *PKL* transcription. This activating function is in clear contrast with the CCA1 repressive role of core clock genes. CCA1 also regulates the expression of *HAF2* (*HISTONE ACETYLTRANSFERASE OF THE TAFII250 FAMILY 2*). HAF2 seems to promote H3ac at the *PRR5* and *LUX* promoters to activate their expression [140].

Two reports also showed a connection of JMJD5/JMJ30, a putative histone demethylase Jumonji C (JmjC) protein, with the plant circadian clock [141, 142]. *JMJD5/JMJ30* has a peak of expression in the evening, a pattern of expression that is regulated by direct binding of CCA1 and LHY to the *JMJD5/JMJ30* promoter to repress its expression [141]. JMJD5/JMJ30 in turn promotes *CCA1* and *LHY* expression and consequently *jmjd5/jmj30* mutant plants display a short-period circadian phenotype [141, 142]. However, overexpression of *JMJD5/JMJ30* also leads to short-period circadian phenotypes [141], which raises the question of the JMJD5/JMJ30 mechanism of action within the clock. Notably, a short-period phenotype was also found in *jmjd5* mutant mammalian cells, and both orthologs were able to lengthen circadian period when expressed in the reciprocal system [142]. These results suggest a similar function of JMJD5/JMJ30 in plants and mammals.

The clock component CCA1 has also been associated with other chromatin-related factors. For instance, MUT9P-like-kinase 4 (MLK4), a kinase that phosphorylates histone H2A at S95, directly interacts with CCA1 and this interaction permits MLK4 to bind to the promoter of the clock- and flowering-related gene *GIGANTEA* (*GI*) [143]. CCA1 also interacts with a subunit of the Swi2/Snf2-related ATPase (SWR1) and NuA4 complexes [143]. These complexes participate in the deposition of the histone variant H2A.Z and histone H4 acetylase activities, respectively. Mutation of MLK4 results in decreased *GI* expression, which correlates with reduced histone 2A serine 95 phosphorylation (H2AS95Ph), H2A.Z, and histone 4 acetylation (H4Ac) at the *GI* locus. The regulation seems to be important for flowering as *mlk4* mutant plants flower late [143]. Notably, ELF3 also co-immunoprecipitates with MLK1–4 [144] and analyses of *mlk1–4* loss-of-function mutants showed an alteration of circadian period [144]. It would be interesting to fully uncover the relevance of MLK1–4 interaction with ELF3. CCA1 and LHY also interact with the LSD1-like histone demethylases LDL1 and LDL2 to repress *TOC1* and likely other CCA1 gene targets [145]. LDL1 and LDL2 also interact with the histone deacetylase HDA6 so that they coordinately control histone demethylation and deacetylation at the *TOC1* locus [145]. Thus, HDA6 not only interacts with PRR9 and TPL but also with CCA1 and LHY. Yeast two-hybrid assays have shown that CCA1 also interacts with the deacetylase SIRT1 (SIRTUIN1) [138]. These results are interesting as mammalian SIRT1 interacts with the core clock component CLOCK to regulate its chromatin-related function [146]. However, further experiments are necessary to fully confirm the CCA1–SIRT1 interaction in plants and to demonstrate the biological relevance of such an interaction.

## Chromatin and clock interplay in *Arabidopsis* hybrids and other crops

Circadian regulation is highly conserved among flowering plants, and the function of central clock genes in *Arabidopsis* can be complemented by homologous genes in rice and maize [147, 148]. Changes in plant growth and development in response to adaptation and selection could have an epigenetic basis [149]. Natural variation of circadian clock features contributes to plant fitness over a wide geological spectrum [150], although the genetic and/or epigenetic basis for altered clock parameters is unclear. In addition to their sessile nature, plants have plasticity in their genomes that can be reprogrammed through hybridization and polyploidy, providing a pervasive force in the evolution of eukaryotic genomes [151, 152]. In *Arabidopsis suecica*, a naturally formed allotetraploid and its resynthesized siblings, expression waveforms (or amplitudes) of circadian clock genes (e.g., *CCA1*, *LHY*, *TOC1*, and *GI*) are altered because of histone acetylation and methylation changes presumably resulting from intergenomic interactions between the hybridizing parents [153]. As a result, the circadian-mediated output regulatory pathways, including photosynthesis and starch metabolism, stress responses, and phytohormonal production, are rewired in response to the clock change [154]. The more starch is produced during the day [153], the more can be degraded and utilized at night [155] to promote plant growth. However, it is unclear whether epigenetic modification of circadian clock genes is a result of interspecific hybridization or the cause of the altered circadian regulatory networks. It is also notable that expression waveforms (or amplitudes) of the circadian clock genes are changed, while the diurnal or circadian period is maintained in these examples to sustain growth vigor.

In an ever-changing environment, plant growth is also influenced by other factors including defense to biotic and abiotic stresses. This trade-off balance is mediated through the internal circadian clock that regulates expression of biotic and abiotic stress-responsive genes [156]. Under normal growth conditions, parents have a memory to elevate expression of stress-response genes, which is inherited from their adaptation to local environments [97, 157]. This stress-response memory is erased and reprogrammed in *Arabidopsis thaliana* hybrids by expression changes in the circadian clock genes through epigenetic mechanisms to save the energy from defense to promote growth [97]. Under stress conditions, however, expression of stress-responsive genes from both parents is inducible at certain times and in certain stress environments, depending on the type of biotic or abiotic stress, for defense, which could have minimized the energy cost, compared with constitutive expression of stress-responsive genes in their parents, of defense for growth [97]. When the stress-responsive genes cannot be epigenetically suppressed in the hybrids, they suffer from hybrid vigor to cause hybrid weakness [158]. Moreover, the circadian clock regulates expression of many other genes involved in biosynthesis and signaling of phytohormones, including auxin and ethylene [159, 160]. Diurnal downregulation of ethylene biosynthesis genes in hybrid plants could also lead to growth vigor; however, the regulation of ethylene biosynthetic genes by CCA1 is indirect [161], suggesting involvement of other factors such as epigenetic ones and other clock components.

There is evidence that expression of circadian clock genes is affected by DNA methylation through the RdDM pathway [162]. In the RdDM gene mutants *ago4* and *nrpd1*, CHH methylation levels in the *CCA1* promoter region are reduced, while the *CCA1* expression waveform is increased. This change in DNA methylation is associated with the parent-of-origin effect on *CCA1* expression in the hybrids, as if *CCA1* expression is imprinted by the RdDM pathway, which is consistent with maternal expression of *NRPD1* in *Arabidopsis* [163]. However, we do not know how DNA methylation controls *CCA1* expression or overall circadian rhythms. *CCA1* expression is not altered in the maintenance methylation mutant *met1* or *ddm1* [162]. Methylome analysis in 3-h time intervals does not seem to support an overall diurnal rhythm of DNA methylation in *A. thaliana* (unpublished data).

Cotton fiber development is influenced by seasonal changes, probably because of temporal regulation in different growth conditions during the winter and summer [164]. This change is coincident with CHH methylation changes in the promoters of some fiber-related genes, which is confirmed by reducing *ROS1* expression in the transgenic cotton, promoting fiber growth in the summer. The role of DNA methylation in seasonal variation hints at a connection with the circadian clock, but it is unclear if the methylation variation affects expression of circadian clock genes in cotton.

Flowering time in plants is controlled by the photoperiod pathway that involves CONSTANS (CO) and flowering locus T (FT), which are regulated by the circadian clock and light signaling pathways [165]. In *Arabidopsis*, overexpressing *CCA1* delays flowering [27], while the *cca1* mutant has an early flowering phenotype [166]. In sorghum, pseudoresponse regulator 37 (PRR37) activates *CO* and represses *FT* in the long-day condition, causing late flowering, and consequently, the mutant *prr37* has an early flowering phenotype [167]. Similarly, *PRR* and *GI* homologs are associated with flowering time quantitative trait loci in rice, maize, and other crops (reviewed in [168]). Although epigenetic regulation of vernalization and flowering time has been extensively investigated [169], little is known about the epigenetic link with circadian regulation in control of flowering time. A recent study demonstrated that some key regulators controlling photoperiodic flowering, such as *CO* or *CO-Like* (*COL*) genes, are among the epialleles that are generated during allotetraploid cotton evolution [170]. *GhCOL2* is methylated and silenced in the wild relatives and hypo-methylated and expressed in the cultivated cotton. Reducing *COL2* expression in the cultivated cotton delays flowering. The result suggests a role for epialleles in the circadian-mediated pathway that regulates flowering time and shapes crop domestication.

## Future directions

The studies summarized in this review clearly establish that the plant circadian clock is directly connected with chromatin modifications. Despite the wealth of information on the interactive interplay between chromatin components and circadian regulators, we are still far from a complete understanding of the molecular and cellular basis underlying this connection. Many questions remain to be answered. For instance, how do environmental cues trigger the clock–chromatin interactions, spontaneously or in a sequential manner? The diurnal fluctuations in light and temperature correlate well with oscillatory patterns of histone modifications at clock loci. However, it is not fully known whether the clock gates specific chromatin signatures in response to fluctuating environmental stresses. Similarly, does the stress-dependent transcriptional activation or repression of clock genes depend on gated chromatin changes? Is it possible that the gated chromatin signatures provide a memory of recent transcriptional activity? Addressing these questions is pertinent in the context of climate change and global warming, which impose a real threat to agricultural productivity. Based on the role of the circadian clock in plant responses to stresses, a full understanding of the environmental factors coordinating the chromatin and transcriptional landscapes would be critical to improve plant fitness and productivity.

The intricate connection between the circadian oscillations and chromatin modifications also opens a key unresolved question about which one is the “cause” and which one is the “consequence”. It is known that circadian clock components and chromatin regulators form functional protein complexes that correlate with changes in circadian gene expression, DNA methylation, and chromatin modifications. However, it remains to be defined whether circadian clock components recruit the epigenetic factors to genomic targets for circadian output or the epigenetic modifications facilitate the recruitment of clock and other factors for circadian regulation. Answering this question is not trivial but it will provide key information about how the epigenetic and circadian transcriptional landscapes are temporally coordinated. In addition, spatial coordination of circadian and chromatin regulation is important to plant growth and development. Research is rapidly and significantly advancing our understanding of how the clock works in different cells and tissues and within the whole plant. The cell and tissue specificity of the circadian transcriptional landscapes might very well be correlated with similar spatial specificities of chromatin remodeling. It is possible that specific chromatin components and marks connected with clock loci only function at particular cells or tissues depending on the specificities of clock outputs on those cells and tissues.

Another interesting aspect that remains to be fully explored is the evolutionary trajectory of clock and chromatin remodeling. From the initial studies in the model system *A. thaliana*, research is increasingly advancing in analyses of clock and chromatin function in other non-model plants. The use of multidisciplinary approaches, including chronobiology, chromatin biology, mathematical modeling, and molecular evolution, will help us to define the similarities and differences across the plant kingdom over evolution. These studies will also provide information on how the circadian clock function is able to regulate the physiological and developmental diversity of different plants such as monocots and eudicots. Lastly, the development of new tools and integrative methods, including but not limited to chromatin and transcriptomics profiles at the single-cell level, will further uncover the intrinsic complexity of chromatin and circadian regulatory networks at both cellular and organismal levels.

## Abbreviations

CCA1: CIRCADIAN CLOCK ASSOCIATED1
ELF: EARLY FLOWERING
FACT: Facilitates chromatin transcription
H2Bub: Histone 2B ubiquitination
H3K27ac: Histone 3 lysine 27 acetylation
H3K36ac: Histone 3 lysine 36 acetylation
H3K36me2: Histone 3 lysine 36 dimethylation
H3K4me3: Histone 3 lysine 4 trimethylation
H3K9ac: Histone 3 lysine 9 acetylation
H3S28ph: Histone 3 serine 28 phosphorylation
HAT: Histone acetyltransferase
HDAC: Histone deacetylase
HMT: Histone methyltransferase
LDL: LSD1-like histone demethylase
LHY: LATE ELONGATED HYPOCOTYL
LNK: NIGHT LIGHT-INDUCIBLE AND CLOCK-REGULATED
LSD: Lysine-specific demethylase
MLK: MUT9P-LIKE-KINASE
PRR: PSEUDO RESPONSE REGULATOR
RVE: REVEILLE
SDG: SET domain group
SIRT1: Sirtuin1
TE: Transposable element
TOC1: TIMING OF CAB2 EXPRESSION1
